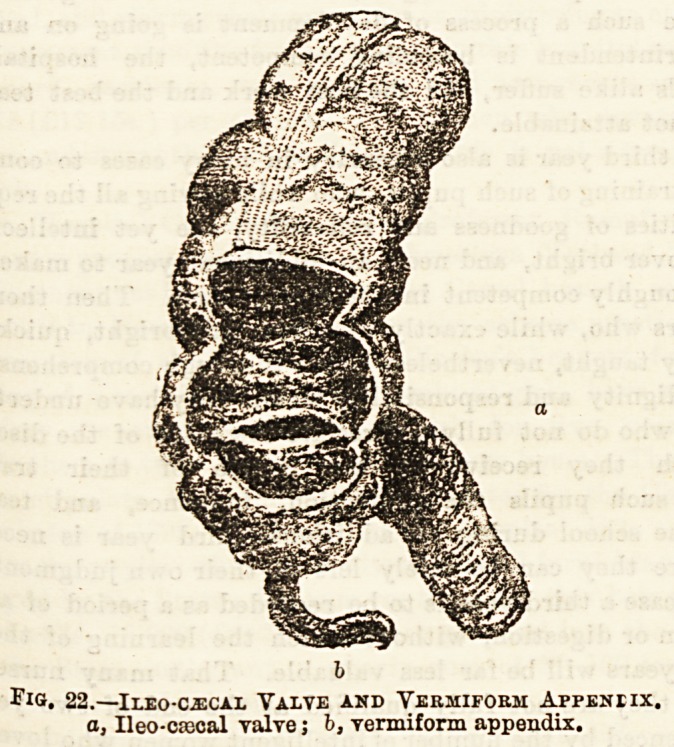# The Hospital Nursing Supplement

**Published:** 1895-04-20

**Authors:** 


“The Hospital” Nursing Mirror.

Being the Extra Nursing Supplement of “The Hospital” Newspaper.

[Contributions for this Supplement should be addressed to the Editor, The Hospital, 428, Strand, London, W.C., and should have the word “Nursing” plainly written in left-hand top corner of the envelope.]

### News from the Nursing World.

### EAST AND WEST.

The funds of the East London Nursing Association and the Parochial Mission Women Fund ought to derive substantial benefit from the Amateur Art Exhibition, which is to be opened on May 6th by H.R.H. the Duchess of York. On the second day Princess Christian has promised to open the exhibition, and the same office will be performed on the 8th by Princess Mary Adelaide. The Working Ladies' Guild and the Guild of Handicraft will be represented, and Mr. S. H. Morley's School of Carving; pictures, sculpture, and art work (including leather and silver) will also be exhibited. A loan of over a hundred specimens of miniatures by Richard and Maria Cosway forms an interesting feature of the show, to which Royal patronage has been so generously promised.

### PRINCESS LOUISE.

The Countess Cadogan has consented to open the bazaar arranged to take place, under the patronage of H.R.H. the Marchioness of Lorne, in June next, at Queen's Gate Hall, South Kensington. The bazaar has been organised to raise funds for sending sick children to the seaside or to the country, and it will last for three days, commencing June 27th.

### UNAUTHORISED COLLECTIONS.

Charles Godby, a waiter, who pleaded guilty at the recent Bristol Quarter Sessions, of obtaining money under false pretences, has been sentenced to twelve months' imprisonment with hard labour. He represented himself as a collector for the local General Hospital, and also for a nurse there, who, he stated, desired to go abroad. The kindly feeling towards nurses which exists among the public amply showed itself in the willingness with which the charitable gave money to an unknown man for the benefit of a well-known nurse. The subsequent exposure of the imposture should emphasise the disadvantages of indiscriminate almsgiving compared with the advantages of aiding deserving and disabled nurses by means of subscriptions to “The Hospital Convalescent Fund,” or to “The Junius S. Morgan Benevolent Fund.”

### WOMEN AND SANITATION.

“Women's Work in Sanitary Reform” is one of the subjects announced for discussion in the coming Sanitary Conference at Manchester. Mrs. Hardie will give an account of the work of the Manchester Ladies' Health Society, of which she is president. The Conference commences on the 24th inst. and lasts for three days, and should prove of general interest.

### DIFFICULTIES LIGHTLY MET.

A new light was last week thrown on the “Cottage Nurse” question by the writer of an interesting little article in a medical contemporary. From it we learn that although the Dorset Health Association considers a certain written examination desirable for cottage nurses, yet this “may be omitted if the candidate has difficulty in reading or writing.” Surely, this unique way of dealing with a preparatory qualification for sick nursing can hardly be consistently maintained when the label of the medicine bottle or the directions for outward applications are in question? In vain too will the country doctor leave those written instructions by which he knows that a trained district nurse can be accurately guided in carrying out his treatment. For the woman to whom reading and writing present difficulties some calling in which they are altogether unnecessary had better be chosen.

### LADIES AT CHELMSFORD.

A committee has been appointed by the Chelmsford Guardians to inquire into the nursing at the infirmary. Hitherto there has been no regular night-nurse, one trained nurse and an untrained under-nurse having charge of from 80 to 100 patients by day, besides dividing the night duties when needful. There being an average of one death per week in the wards it is obvious that some patients must always be in need of skilled attention. The three lady guardians have done well to call attention to the over-working of the nurses and to the need for modification of existing arrangements.

### A SHORT-SIGHTED POLICY.

Certain Lancashire men have failed to maintain their local reputation for shrewdness by their recent action respecting a head nurse for the Lancaster Workhouse. For the advertised vacancy eight candidates applied, and, after careful consideration, the committee decided that not one was competent to fill the post, which is one of much responsibility. They recommended that the advertisement should be repeated, and £25, instead of £20, per annum offered to a suitable person. Although this suggestion was supported by the Mayor, it was negatived, and the Guardians finally agreed that the original advertisement should be repeated. The ardour of the commiteee in attempting to improve the nursing department must be somewhat damped by so marked a slight being put upon their recommendations. Lancashire men ought to place a higher value on such skilled work as that done by the trained nurse.

### NURSES AT LEICESTER.

The annual meeting of the Leicester Institution of Trained Nurses, which was held last week, received the first report issued since the separation of the district from the Private Nursing Branch. The work of both departments has been successful, although the private nurses have not been fully employed, owing to the unusual good health in the neighbourhood during the year. The financial position is, therefore, not quite as satisfactory as could be wished for. The Bishop of Peterborough was in the chair, and the meeting was well attended.

### QUEEN'S NURSES IN WALES.

The annual meeting of the Welsh branch of the Queen's Jubilee Institute, recently held at Cardiff, was an extremely satisfactory one. In North Wales ten associations of district nurses have become affiliated, and twelve in South Wales, and the record of work is excellent. Steady progress has characterised district nursing in the Principality, in spite of the ever-present difficulty of getting an adequate supply of Welsh-speaking women. It having been suggested that the report dealt too much with the nursing at Cardiff, to the exclusion of the affairs of the affiliated branches, it was proposed and carried that in future the presidents and secretaries of the local branches should be on the council of the Welsh branch of the Institute, which is a very influential and representative body.

### A FRENCH HOSPITAL SCHOOL.

A new hospital is in course of erection in Paris, which will bear the name of the Auteuil Hospital, and will cost some £18,000. It is designed by the Association des Dames Françaises to ensure practical training for the members of the society in the care of the sick. To carry out this training, a ward is to be furnished for twenty-four patients, and no doubt every facility will be given to the ambulance pupils to learn the elements of nursing.

### A CANCER HOSPITAL.

"A cancer hospital? No, thanks, I'd rather not!" replied a friend invited to join an expedition to the Fulham Road. Many share the feeling that there must necessarily be something dreadfully sad in such a place; yet, in reality, there are no more cheerful wards in London than those through which the stranger is courteously conducted at Fulham, and certainly in no country in the world are there better kept ones. The polished floors are spotless, and the tiled walls, the staircases, and picture-frames are marvellously free from dust. The snowy quilts and bright spring flowers seem fitting accompaniments to the cheerful, hopeful occupants of the wards. Like their consumptive neighbours at the Brompton Hospital, these cancer cases are largely endowed with hopefulness, and there is little to show the casual visitor that many patients are grievously afflicted. In the prettily laid-out grounds a large number pass the long summer days, and a fine "garden-room" offers shelter from keen winds or fierce sun. Another room in the grounds is given up to the smokers, for whom games and papers are also provided. In the winter, concerts and dramatic performances take place, and in the summer they are replaced by garden parties. Not the least pleasant feature of the hospital is the nursing staff, whose personal neatness, particularly in the matter of caps and hair, might well be copied by many probationers to whom the interior of a cancer hospital is an unexplored territory.

### HOMES AT ALEXANDRIA.

A proposal to rebuild the Victoria House and Nurses' Home at Alexandria was considered at a general meeting recently held under the presidency of Sir Charles Cookson, H.B.M.'s Consul-General. The house was originally taken to receive governesses and other lady workers at three francs a day, and women of the domestic servant class at one and a half francs. It has been found that the first are far the most numerous supporters of Victoria House, and therefore when the new home is built it will provide only for them, several other establishments being now open to servants in Egypt. Associated with Victoria House there is a separate nurses' home, which appears to be considered a distinct benefit to visitors and residents.

### BELLEVUE TRAINING SCHOOL, U.S.A.

At the request of the Commissioners of Charities, the charge of the Female Insane Pavilion was last year accepted by the managers of the Bellevue Training School, the number of nurses on the staff being raised to sixty-six. Lectures are given by doctors on hygiene, physiology, symptoms of disease, anatomy, surgery, obstetrics, massage, and electricity, and every week classes are held on cooking for the sick. The doctors' lectures are supplemented by class instruction given by Miss Bennan, the superintendent, and her assistants. The course of training covers two years, but only the first is entirely spent by the probationers in the wards, as they are engaged in district and private nursing during the second. At the conclusion of the term of training a final examination is held, and successful candidates obtain a diploma signed by the examining board and by the committee of the board of managers. A post-graduate course was attended last year by some of the older nurses, who fully appreciated the opportunity of acquainting themselves with the newest methods and appliances.

### MEDICAL AID FOR WOMEN OF INDIA.

At Calcutta last month the annual meeting in connection with the Countess of Dufferin's Fund way presided over by Lord Elgin. He said that many of the hospitals which the Fund had been instrumental in establishing had been recently inspected by Lady Elgin, and he was able to assure his hearers that the reluctance of native women to enter these institutions was steadily decreasing. A patient who personally experienced the care and skill there placed at her service was usually quite willing to return to the ward if her health rendered further treatment desirable. Some particulars of the aims of this Fund appeared in "The Nursing Mirror" of March 30th.

### SHORT ITEMS.

The Rugby Guardians have secured the services of a trained and experienced nurse through the Workhouse Infirmary Nursing Association, at a salary of £30 per annum.—Miss Power, the present matron at Cromwell House, Highgate, has been appointed matron of the new Convalescent Home at Broadstairs.—The engagement of a trained nurse for the sick wards at Conway Workhouse will be discussed at the next meeting of Guardians.—Nurse Sarah Collings has tendered her resignation to the Honiton Guardians, at the age of 75, after thirty years faithful service in the workhouse.—There are two convenient rooms at the Trained Nurses' Club, 12, Buckingham Street, Strand, which can be hired for meetings, classes, &c., at very moderate terms, many people would repeatedly use so central a place if they had once paid it a visit.—The annual report of the Sunderland Nursing Institute has been issued, and contains the rules for the nurses and probationers, and details of their duties to the sick poor and to private patients.—It is announced that the annual meeting of the Northern Workhouse Nursing Association will take place at the Lincoln Masonic Hall on the 19th inst.—Nurse Woolnough was presented with a gold watch at the conclusion of her course of lectures on "Domestic Economy" at Glasgow.—The Nottingham Board of Guardians are about to appoint six additional nurses for their workhouse.—Miss Olive Walters has been promoted to the post of Staff Nurse at Greenwich Union Infirmary; she obtained the second prize at the recent annual examination.

### Elementary Anatomy and Surgery for Nurses.

By W. McAdam Eccles, M.B., M.S., F.R.C.S., Lecturer to Nurses, West London Hospital, &c.

XIII.—THE DIGESTIVE SYSTEM (*continued*).

The small intestine consists of three portions, (1) the duodenum, (2) the jejunum, and (3) the ileum (see Fig. 21 in last issue). The duodenum is about ten inches, or twelve fingers breadth, in length. It begins at the pylorus on the right side of the spine, and, describing a horse-shoe curve, crosses to the left side about the level of the second lumbar vertebra where the jejunum commences. This curve embraces the head of a gland called the pancreas. The jejunum and ileum together form a much convoluted tube some twenty feet in length. This hangs from a fold of peritoneum, which is attached to the posterior wall of the abdomen and is termed the mesentery; the small intestine is thereby comparatively freely movable, but the coils are in close contact with each other and the abdominal wall, only a potential space intervening in the normal state.

The large intestine, about six feet long, is again divided into several portions, which are termed respectively (1) the cæcum, (2) the colon, and (3) the rectum. The cæcum is a blind pouch situated on the right side of the abdomen near

Fig. 22.—Ileo-cæcal Valve and Vermiform Appendix. *a*, Ileo-cæcal valve; *b*, vermiform appendix.

the iliac bone, and below the junction of the ileum with the large intestine. From its lower part springs a narrow worm-like tube some three or four inches in length, called the vermiform appendix. (See Fig. 22.) At the spot where the small intestine joins the large are two folds of mucous membrane, forming what is spoken of as the ileo-cæcal valve, which serves in great measure to prevent regurgitation of the contents of the colon into the ileum. (See Fig. 22.) The colon may be said to consist of four parts, the ascending, transverse, descending colon, and the sigmoid flexure. These portions form three sides of a square, and their relative positions are well shown in Fig. 21. The alimentary tract terminates in the rectum, and opens externally at the anus.

The associated glands of the digestive system, besides those which are found in the mucous membrane of the tube itself, are: (1) The salivary, (2) the liver, (3) the spleen, and (4) the pancreas.

The salivary glands secreting the viscid fluid found in the month and called the saliva are three on each side, the parotid, the submaxillary, and the sublingual. The parotid glands are situated in front of and below the ears, and are the largest of the salivary glands proper. The duct of each opens into the mouth opposite the second upper molar tooth. Inflammation of these glands constitutes mumps. The submaxillary glands lie beneath the lower jaw, one on each side. The ducts open on the floor of the mouth beneath the tip of the tongue. The sublingual glands form ridges below the tongue, and each has several ducts which for the most part open separately into the mouth beneath the tongue.

The liver is the largest gland in the body, weighing no less than fifty to sixty ounces in the adult. It is placed in the upper part of the abdomen, close under the diaphragm, and mostly on the right side. There are two chief lobes, the right the larger, the left the smaller. The upper surface, which is smooth and convex, fits accurately the under concave surface of the diaphragm. The lower surface is irregular, and presents certain fissures, smaller lobes, and the gall bladder as well as the vessels entering and leaving the liver. The blood from the stomach, intestines, spleen, and pancreas containing much of the digested and absorbed food, reaches, by means of a large vein named the portal, the transverse fissure of the liver, where the vein, dividing into two branches, breaks up into minute vessels or capillaries within the liver substance, to again become collected into two other large veins, the hepatic, which themselves open into the vein which passes through the diaphragm to empty into the heart. In addition to this the liver also derives blood from the hepatic artery. The organ secretes bile, which is conveyed away from it by two hepatic ducts, and is partly stored in the gall bladder, and partly passes into the duodenum by the common bile duct.

The spleen is a dark purple-grey organ, and is not, strictly speaking, a gland, for it has no duct. It weighs about six ounces, and has an outer convex surface in contact with the diaphragm, and an inner surface in relation with the stomach and left kidney.

The pancreas lies behind the stomach across the spine. It presents a head, body, and tail. Its ducts open with the common bile duct into the duodenum. It secretes a fluid somewhat of the nature of saliva, called the pancreatic juice.

### Appointments.

[It is requested that successful candidates will send a copy of their applications and testimonials, with date of election, to The Editor, The Lodge, Porchester Square, W.]

Royal Hospital for Children and Women.—Miss F. Beatrice Hunter, who was trained at King's College Hospital, has received the appointment of Matron at the Royal Hospital for Children and Women, Waterloo Bridge Road. We wish her every success in her new work.

Royal London Ophthalmic Hospital.—Miss Ada Robinson has been appointed Lady Superintendent of this hospital. She was trained at the London Hospital, where she has held the posts of holiday and ward sister. Miss Robinson has also had considerable experience in housekeeping, general management, &c., and is well qualified for the position which she has been chosen to fill. We wish her continued success in her new work.

Kimberley Hospital, South Africa.—Miss Gertrude Vacher has been appointed Matron of this Hospital. She was trained at the London Hospital, where she also held the position of staff nurse. Miss Vacher was afterwards matron of Eccles Hospital, and did excellent work there, and subsequently became matron of the Accident Hospital at Poplar. We congratulate the authorities at Kimberley on having secured for their hospital a lady whose training, experience, and ability specially fit her for filling a responsible position with distinction. Many good wishes from old friends will accompany Miss Vacher to her new home.

### Nursing in America.—The Convention of Superintendents and Members of the Association.

### THE THREE YEARS' COURSE OF TRAINING IN CONNECTION WITH THE EIGHT HOURS SYSTEM.

By Mrs. Hunter Robb (Isabel Hampton), late Superintendent of Nurses at Johns Hopkins Hospital.

Some time over a year ago it was my privilege to prepare for the International Congress of Charity and Correction a paper dealing with the standards of education to be demanded of nu'ses both before and after their entrance into a training school. It may be remembered by some of you who are now present that I spoke at some length of the necessity of a careful elimination of the undesirable candidates who present themselves. I insisted that not every woman who desires to take up the profession of a trained nurse has the natural capabilities, or has had the educational advantages which are necessary to such a career. But I pointed out that, after obtaining suitable material, it is necessary to make the best possible use of it, and that here the second part of our duty begins.

Among other changes advocated in the paper just referred to was the extension of the course over a period of three years, with a day of practical work consisting of eight hours. At that time the reasons for these changes, and suggestions as to the manner in which they could be carried out, could be only broadly outlined. The object of the present paper is to consider these reasons in detail, and try to arrive at some practical conclusion which will facilitate the establishment of such a course in the various training schools.

The subject should be dealt with without bias for any school in particular, but with a view to the best interests of all training schools which are able to undertake satisfactorily the important duty of training nurses. Between these schools there should exist a spirit of unity, and it should be our earnest desire to establish a standard of education that will be common to all. To bring about this should be, and I believe is, one of the chief aims of our association. And it seems to me that just at present no better opportunity could be afforded to us to accomplish our end than in uniting in developing the three years' course of instruction, and in agreeing, after due discussion, upon the adoption of some scheme which should also include (1) specifications of the necessary qualifications of applicants; (2) a curriculum for teaching and study; and (3) a proper grading in tests and in final examinations for certificates.

That some extension of the period of training is generally desired was evidenced by the informal discussion of the subject that took place in this assembly last year, by the suggestions since offered by the writers in our various magazines devoted to the subject of nursing, and by the fact that since the International Congress some one or two schools have lengthened their course so as to make it extend over three years, while others have this step under serious consideration.

A superintendent of a training school owes a duty, first to the hospital, and secondly to the nurses under her. These duties are of equal importance, the hospital must not be sacrificed, but neither have we any right to sacrifice the wellbeing of our nurses; some scheme must be adopted which will prove advantageous to both. I shall therefore consider a little in detail the advantages or disadvantages to the hospital and to the nurses which may result from the adoption of the plan suggested. For the hospital the advantages are readily seen. In the first place, the hospital would have better nurses, since it would be benefited by having more experienced nurses during the third year of their course. Again the hospital and training school would be relieved of the disadvantages of having to deal with so much raw material at such frequent intervals, and the school would be enabled to select from the candidates much more closely, and thus a higher standard could be more easily obtained.

If the third year's instruction were made to include a course for nurses who wished to prepare themselves more especially for hospital positions the hospital would again be benefited, because under present conditions superintendents of schools have no opportunity of learning the administrative duties of such a position until after they have undertaken it. Our present methods of training allow but few opportunities for a woman to gain this practical knowledge; hence the success of a new superintendent of a training school must depend upon her native ability, and such stray knowledge as she may have been able to pick up while occupying the position of head nurse. More than one nurse's career as a superintendent has been cut short by mistakes made through ignorance in the beginning of her administration—mistakes which never would have occurred had she had an opportunity beforehand to become practically acquainted with the duties of her new position. Again, it must not be forgotten that while such a process of development is going on and the superintendent is becoming competent, the hospital and pupils alike suffer, and the best work and the best teaching are not attainable.

A third year is also necessary in many cases to complete the training of such pupils, who while having all the requisite qualities of goodness and reliability, are yet intellectually not over bright, and need an additional year to make them thoroughly competent in their profession. Then there are others who, while exactly opposite, are bright, quick, and easily taught, nevertheless lack a thorough comprehension of the dignity and responsibility which they have undertaken, and who do not fully appreciate the value of the discipline which they receive in the course of their training. For such pupils the protection, influence, and teaching of the school during an additional third year is necessary before they can be safely left to their own judgment. In any case a third year is to be regarded as a period of assimilation or digestion, without which the learning of the first two years will be far less valuable. That many nurses feel that they are not fully qualified at the end of two years is evidenced by the number of intelligent women who love their work, and who are interested in their profession, and who beg to be allowed to stay another year. By the establishment of the three years' course it is hoped that the number of such women would be much increased, since we may naturally expect and hope that the commercial woman will be excluded by the adoption of this plan; and even if we have fewer graduate nurses they are much more likely to be competent, and after all this is the main point. As a matter of fact a slight diminution in number would not be an altogether unmixed evil. Just now the number of graduate nurses engaged in private nursing is, I am told, so great, and is growing so rapidly all the time that many nurses are without patients half the year. I am informed that in the city of Philadelphia there are so many that a committee of physicians have already held a meeting in order to discuss the possibility of taking advantage of this condition in order to reduce the remuneration for the services of graduate nurses. A somewhat unwarrantable proceeding on their part it would seem. But if this question is to be regulated by the laws of supply and demand then a diminution in the number of graduates will insure a lucrative occupation to those who have had a thorough training, and who hold certificates of competency.

*(To be continued.)*

### Nursing in Germany.

(CONTRIBUTED.)

THE authorities of the Royal Charité Hospital, in Berlin, have ordered the provision of a special ambulance fitted with indiarubber wheels, which, at any moment of the day or night can be dispatched to the scene of an accident on receipt of a telegram or telephonic summons. Special notices to this effect have been served to the police of Berlin and neighbourhood, explaining also that no charge is made to the poor. A doctor will accompany the ambulance if needful.

A boarding house has lately been erected at Pistyan, the Hungarian watering-place, under the auspices of the medical authorities of the town, to bring sulphur baths within the reach of the working-classes.

Proposals for the enlargement of the Hamburg Hospital nursing staff have been made since the cholera epidemic of 1892. A complaint is made that too many women undertake nursing as a means of subsistence rather than as an act of charity. They therefore relinquish it as soon as more profitable occupation offers. At the time of the epidemic, when the assistance of medical students and doctors from other towns was sought, the former received a daily gratuity of M. 3 for working night and day in the Eppendorfer Hospital, and risking their own lives in ministering to others. At the present time the female nurses in this hospital receive free board, residence, and uniform, and an average sum of M. 275 (£13 15s.) per annum. For women who devote life service and strength to their fellows, this is not a tempting outlook.

The Hospital Committee, with Professor Rumpf at its head, now proposes to form a "Schwestern Verband," and an agreement has been made with the Cassel branch society to start it in Hamburg. To render the scheme practicable, the executors of the Heinrich Schmilinsky's bequest, for the training of a certain number of Hamburg girls, have agreed to defray the expenses of twenty girls for five years, to enable them to train as nurses in the new home. The committee stipulate that the building granted by the Cholera Commission of 1892, which adjoins the hospital, is to accommodate the pupils; the cost of alteration and furnishing to be defrayed by the State; the sum required for this being estimated at M. 13,000 (£690). Another proposal is to the effect that the State shall grant a yearly sum of M. 100 towards a pension for each sister.

The contemplated change would make slight difference to the hospital finances; at the present time the inclusive female nursing staff is paid the sum of M. 48,749, a society connected with the hospital might possibly raise the expenditure to M. 56,500.

Fourteen nurses have completed their training at the Institute for Jewish Nurses in Berlin, and are doing good work as district nurses, making no religious distinctions among their patients, and they will be shortly joined by others who have almost completed their term of probation. The training they receive is excellent; for all gain practical experience of the nursing of men, women, and children, and also in the infectious wards. They receive theoretical instruction from the two medical directors of the Jewish Hospital, Drs. Israel, and Lazarus. The Jewish "Verein" has in view the erection of a sisters' home, which it is hoped will be completed and opened in the summer of 1896.

### Minor Appointments.

CITY HOSPITAL, PARKHILL, LIVERPOOL.—Miss Mary E. Parker has been made Deputy Matron to the City Hospital, Parkhill, Liverpool. She trained for fever nursing at Monsall, and had her general training at Newcastle Infirmary. She was night superintendent at Stafford and charge nurse at Liverpool City Hospital South until she left to enter upon her new duties. Miss Parker takes many good wishes to her new work.

### Notes from Victoria.

(COMMUNICATED.)

THE unsettled state of Parliament in Victoria for some months past has postponed all legislation upon the charities, nor is anything likely to be done except, perhaps, a further reduction of the grant in aid until the finances are put straight. Meantime all the hospitals and other institutions are suffering from want of funds, the public response to their special appeals being much less in amount than formerly.

Diphtheria and typhoid are both raising their hydra heads again as the summer advances, although, so far, the average of heat is low.

A trained nurse, Miss Selina Sutherland, known all over Victoria, and even further, as the "children's friend," having for the past fourteen years been actively engaged in the philanthropic work of child rescue, in conjunction with a committee of most enthusiastic helpers, has just established a society, under the title of the "Victorian Neglected Children's Aid Society." Miss Sutherland received her training in the Women's Hospital, Melbourne, and took the diploma issued by that institution.

The Central Board of Health is strenuously endeavouring to rouse the various municipalities and shire councils throughout the colony to secure sites for sewage farms, for temporary quarantine grounds in case of an outbreak of small-pox or other infectious disease, and also for burial purposes, before the State parts with the title to the land. All these sites have to be approved by the Board of Health. The Board is also endeavouring to secure the establishment of a system of federal quarantine, and the skilled veterinary inspection of dairy cattle and stock for human consumption.

The returns just furnished by the two coroners, Dr. Youl and Mr. Candler, for the year 1894, show that 381 inquests were held in the city of Melbourne. Drunkenness is held to be responsible for 71 of these. Infanticide (25 cases) shows a decrease, doubtless due to the operation of the Infant Life Protection Act, under which the nursing of children is more strictly regulated than formerly.

Miss Annie Bourke, who has been charge nurse in the Adelaide Hospital, Adelaide, South Australia, for the past nine years, was married on December 29th to a journalist, Mr. William Patterson, son of the Hon. W. Patterson, of Brisbane, Queensland.

Dr. J. MacInerny, of Melbourne, has made complete bacteriological and other preparations for the cultivation of anti-toxin, the new remedy for diphtheria.

An interim vacancy having occurred in the honorary medical staff of the Women's Hospital (midwifery department), the committee of the hospital, instead of filling it up themselves, took the unusual course of having a public election by the subscribers. The election was held on January 11th. There were five candidates, and Mr. George Horne, M.B. (Melbourne University), headed the poll. The term of office will be about nine months, as the ordinary general elections (four years' term) take place in September.

### Death in Our Ranks.

AT Plaistow Nurses' Home, on April 15th, Nurse Hardy died of pneumonia, following measles, after a short illness. She will be deeply regretted by all her fellow-workers and by many other friends.

### Wants and Workers.

[THE attention of correspondents is directed to the fact that "Helps in Sickness and to Health" (Scientific Press, 428, Strand) will enable them promptly to find the most suitable accommodation for difficult or special cases.]

"*Home.*"—Will anyone give the name of a home where a young lady (21), who is almost helpless through rheumatoid, could be received for small payment?—*Hospital Reader.*

### Magazines for the Month.

The Westminster Review for this month has some very interesting articles. "The Evolution of Sex," by J. P. A. Sykes, treats of the much-discussed woman question. Speaking of woman's part in life, the writer says, "If women wish to use their influence in the subduing of present evils, if they desire to raise the moral tone of men's lives, they will work to far greater advantage by employing their subtle wit and keen perception, by retaining the power of their physical weakness and mental strength, their shrinking from vice, and adroitness in amusing, than by inveterate hectoring and probing and petty tilting in the lists on unequal terms, or still more open warfare." There is a charming article on "The Poetry of Christina Rosetti," and an interesting account of "The Jenolan Caves of New South Wales."

The English Illustrated Magazine for April contains, as usual, some delightful short stories; "The Open Shutter," by Stanley Weyman, and "The Encore," by Violet Hunt, are perhaps the best of these. There is an interview with Mr. William Morris, with some account of his work, past and present. The writer tells us, "Among the other books in preparation are selections from the poems of Coleridge and Herrick, the poems of Mr. Theodore Watts, the Romance of Sir Percival, from the Thornton MSS., and a new prose romance, 'Child Christopher.'" These will be valuable additions to the sumptuous publications of the Kelmscott Press.

### Where to Go.

Annual Amateur Art Exhibition.—On May 6th, 7th, 8th, 9th, 10th, Mr. Gretton kindly permits this exhibition to be held at Moncorvo House, Ennismore Gardens.

Morning Concert at Stafford House.—By kind permission of the Duchess of Sutherland, on Thursday, April 25th, at three p.m., in aid of the funds of the Women Lecturers' Association. Tickets can be obtained from Mr. B. Tree, St. James' Hall, and from Miss G. Bradley, 4, Caroline Place, Mecklenburgh Square, W.C.

St. George's Hall, Langham Place.—On May 9th, at eight p.m , Mr. E. Glossop Such will give a dramatic performance in aid of the Siddons Memorial.

Talbotype Gallery, 55 and 56, Baker Street.—An exhibition of the drawings and sketches of the late Sir Oswald Walters Brierly, F.W.S., F.R.G.S., is open at this gallery (Messrs. Elliot and Fry) until May 4th from ten to six. Sir Oswald's work as marine painter to Her Majesty is well known, and the collection will repay a visit. H.R.H. the Prince of Wales has become a purchaser of one of the paintings.

### Novelties for Nurses.

Private nurses may like to know of the "Nurses' Bill and Receipt Book," which Nurse Kathleen Martin has designed for them. Fifty forms and counterfoils are printed to receive in a conveniently condensed shape the statement of the time during which the nurse has been employed at a case; also her travelling and laundry expenses. The book is a satisfactory substitute for loose sheets of paper, and it provides receipts for employers, whilst the counterfoils form a valuable record of the nurse's own earnings, and of the dates at which she has been employed. The book is 1s. 2d. post free, or 1s. if procured at 273, Regent Street, London, from Messrs. Munro and Co.

### For Reading to the Sick.

WAITING.

**Motto.**

They also serve who only stand and wait.—*Milton.*

**Verses.**

The sun wears down it's western slope,
 The lengthening shadows lie,
Like a fast closing gate of hope
 Before the waiting eye
Of idlers in the market place,
 Who now till day's decline
Have sought in vain a master's grace
 In every passing sign.

And on the fierce revolt of powers
 To find their proper aim,
And the hard struggle through the hours
 This unused strength to tame;
The maddening fear that runs it's race,
 Tired hope to overspread!
Poor idler in the market-place,
 Thy story we may read.

But when the Master came 'twas One
 Who knew how sore their day,
That others toiling in the sun
 Had not a harder way;
And each man's hire that day was given,
 In justice fine and true:
Who waits in vain, or who has striven
 Each gains the self-same due.

*Margaret Bradshaw.*

I am no longer eager, bold, and strong,
 All that is past;
I am ready *not* to do
 At last—at last.
My half-day's work is done,
 And this is all my part.
I give a patient God
 My patient heart.

*Found by Miss Alcott under a dying soldier's pillow, in the hospital at Georgetown.*

**Reading.**

Their office was to wait.—*Chronicles.*

Some must suffer and some must serve, but each one is necessary. You are not cut off from the Body of Christ, only taken apart, laid aside, it may be for a season, it may be for life. . . . Your feet may be set fast; they may have run with great activity, and you sorrow now because they can run no more. But do not sorrow thus, do not envy those who are running; you have a work to do; it may be the work of the head or of the eye; it surely is whatever work God gives to you. It may be the work of lying still, of not stirring hand or foot, of scarcely speaking, scarcely showing life. Fear not if He, your Heavenly Master, has given it to you to do, it is *His* work and He will bless it.

—*Adaped from "Sickness, its Trials and Blessings."*

They have gained the higher glory. To those who wait comes a reward not hoped for or expected. The peace which passeth all expectation is theirs at last. Theirs are the soft strains of rejoicing resignation; theirs is a crown if they care to wear it, more glorious than any wreath of the Nemean games; theirs is the golden harp, with which to celebrate the mysterious victory over sorrow and disappointment—the solution of the problem insoluble to the world, the final triumph of love over pain.—"*'Twixt Harp and Crown.*"

God will make clear the purpose; I at least can wait in silence.—*Plumtre.*

### Everybody's Opinion.

[Correspondence on all subjects is invited, but we cannot in any way be responsible for the opinions expressed by our correspondents. No communications can be entertained if the name and address of the correspondent is not given, or unless one side of the paper only be written on.]

### NIGHT NURSING IN WORKHOUSES.

"One Who Knows Colchester" writes: In a recent number of The Hospital the need of a proper night nurse for the Colchester Union Infirmary was pointed out. Although the present may not be a desirable state of affairs, I venture to think that Colchester will compare advantageously with most country unions in nursing. Unless things have altered recently, it cannot be said that sick paupers in that union suffer from neglect in spite of the absence of a trained night nurse, and the fact that the day nurses have sometimes to get up in the night. The appointment of a night nurse was discussed by the guardians, and the advice of the medical officer obtained (the latter is most kind and considerate to the patients), and he gave it as his opinion that at present no alteration was necessary. He pointed out that if they had a trained night nurse the same system of pauper "sitters up" must be adopted, as the nurse could not be in two places at once. He further called attention to the fact that a large number of infirmary patients need little, if any, actual nursing, as in a small country place cases are admitted to the infirmary which in a larger house would be placed in wards set apart for old and infirm cases. The lady guardian who originated the discussion thought that many ladies would be glad to give their services as probationers in return for training; but it seems unlikely that the limited experience of a small workhouse infirmary would content candidates, however useful the nursing of a succession of chronic patients might eventually prove.

[We fail to see the force of the argument that the inability of a night nurse to be in two places at once counteracts her utility. We also think that the old and infirm have a right to skilled nursing by night as by day.—Ed. *T.H.*]

### PRIVATE NURSES.

"M. R." writes: Being a constant reader of The Hospital, I have from time to time seen complaints made by private nurses of not being able to get cases. I have been nursing on my own account for some time, and find no trouble in getting work in good county families at six guineas per month. I never take my meals with the servants. If I do not have them with the family (which I find it somewhat difficult to do on account of my patient), I have them laid in a room by myself. If institutions and hospitals would only take educated women for nurses people would not look down on them and expect them to work as general servants. Nurses do not go through a long period of training merely to fit them for looking after chronic cases or housework.

### THE LAST THING IN BADGES.

Lady Baker writes: The standard for hospital-trained nurses for the Dorset Health Association is the same as that adopted by the Queen's Jubilee, viz, from one to six years' hospital training. All such women are entitled to wear what, I believe, is considered the usual badge of a nurse, the veil, and also a silver medal. This twelve out of the seventeen nurses working under our association are entitled to do. All under that grade (of whom we have at present only six), namely, probationers of six months' or more standing, or local nurses of experience who are able to pass the examination of our medical committee, without which no one may work under our association, are definitely distinguished by not "assuming the veil," and wear only a bronze medal. Each of our nurses of either grade will shortly have a printed certificate, signed by the medical examiners, stating exactly her qualifications and length of training, and whether qualified for general nursing or midwifery. This she will be required to show when requested to the medical man in attendance. Surely hospital nurses are not bent on the extinction of all attention to the sick and aged but their own. It will not redound to their credit to endeavour to make the profession a close one, and tyranny is sometimes succeeded by a revolt from a long-suffering public. Even our medical men are beginning to ask whether nursing is really such an occult science as some nurses are apt to maintain, and if their country patients are to be allowed to die because the services of a trained nurse at £80 or £100 a year are not available? As long as an association holds to the principle that all nursing must be done absolutely under medical supervision, and ensures that no nurse shall pretend to qualifications which she does not possess, we think the public safety is tolerably well guarded, and that the criticism of your valuable paper might be reserved for more dangerous abuses than those countenanced by the Dorset Health Association. I may add that I have met with the most cordial co-operation on the part of the trained nurses in Dorset, as well as on that of the medical men, in my endeavours to provide them with additional assistance in carrying out their arduous duties.

[We are glad to find that the necessity for training is so far acknowledged by the Dorset Health Association that it employs twelve qualified nurses out of a total of seventeen. —Ed. *T.H.*]

### Notes and Queries.

### Queries.

(119) *Dispensing.*—Can you advise me how to get instruction in dispensing in return for my services? I have done three years' nursing in an incurable hospital, and cannot afford to pay high fees.—*E. E. E.*

(120) *Nurses' Quarters.*—When nurses are provided with separate bedrooms, a general sitting-room, a dining-room, and a business-room, should they demand a second sitting-room as a right? This is a small establishment, and the nurses are permitted to take female friends to their own rooms and to receive male friends in the business-room. The matron also lends her own sitting-room on occasions when a nurse asks leave to see her minister or guardian there.—*One who Desires to be Just.*

(121) *Male Attendant.*—Is there any hospital or asylum where a young man can obtain training in nursing?—*R. R.*

(122) *Training.*—Should I get best training in nursing in a London private nursing home or under the Red Cross system?—*Perplexed One.*

(123) *Canada.*—I shall be glad to know whether English hospitals differ from Canadian ones? I was for several months under a trained nurse in a cottage hospital in Canada, but no certificates were granted there. I want to learn whether certificates are given in all English hospitals?—*J. W.*

(124) *Dye.*—Can you tell me of a safe preparation for bleaching grey hair?—*M. E. C.*

(125) *Voluntary.*—Is there a home, institution, or district where a lady training in midwifery, monthly nursing, and preparing for L.O.S. exam. in July, could give her services from nine until six in London?—*Nurse A.*

### Answers.

(119) *Dispensing* (*E. E. E.*)—You should read the chapter on "Dispensing" in Burdett's "Hospital Annual." You might find it possible to make some arrangement at a cottage hospital where the matron does the dispensing, and the committee might sanction your being received as a probationer. We should, however, strongly advise you to wait until you can afford to pay for a proper course of instruction. You are young enough to wait.

(120) *Nurses' Quarters* (*One who Desires to be Just*).—For a small home you certainly allot ample quarters to your nurses. We do not see what more can be required of you, a nurses' home does not exist primarily for the reception of callers. It is occasionally convenient for nurses to receive visits from their friends, but it is far better, whenever possible, for them to go out. A short change to different surroundings is mentally and physically refreshing to a worker, and therefore should always be encouraged rather than the reception of guests in the off-duty time, originally granted with a view to air and exercise.

(121) *Male Attendant* (*R. R.*)—There is no general hospital in England where such training is given, but in some asylums a young man of good character, mentally and physically suited to the work, can obtain systematic training. Write to the Medical Superintendent of one of the large public asylums, and ask for form of application.

(122) *Training* (*Perplexed One*).—We should advise a full course of training at a general hospital or infirmary. No private nursing institution ranks as a nurse training school.

(123) *Canada* (*J. W.*)—In no hospital would you be entitled to a certificate at the end of a few months. You had better enter a good general hospital, where there are over a hundred beds, in England or Scotland. In applying to a Matron for the rules, you should ask what kind of certificates are given in each hospital.

(124) *Dye* (*M. E. C.*).—We recommend you to trust time and nature to work the desired change.

(125) *Voluntary* (*Nurse A.*).—We do not understand your question. If you are training for a special branch of work, how can you give nine hours a day to anything else? Please explain further.

## Figures and Tables

**Fig. 22 f1:**